# Development and validation of hub genes for lymph node metastasis in patients with prostate cancer

**DOI:** 10.1111/jcmm.15098

**Published:** 2020-03-04

**Authors:** Ning Xu, Shao‐Hao Chen, Ting‐Ting Lin, Hai Cai, Zhi‐Bin Ke, Ru‐Nan Dong, Peng Huang, Xiao‐Dong Li, Ye‐Hui Chen, Qing‐Shui Zheng

**Affiliations:** ^1^ Department of Urology The First Affiliated Hospital of Fujian Medical University Fuzhou China

**Keywords:** hub genes, lymph node metastasis, prostate cancer, weighted gene co‐expression network analysis

## Abstract

Lymph node metastasis is one of the most important independent risk factors that can negatively affect the prognosis of prostate cancer (PCa); however, the exact mechanisms have not been well studied. This study aims to better understand the underlying mechanism of lymph node metastasis in PCa by bioinformatics analysis. We analysed a total of 367 PCa cases from the cancer genome atlas database and performed weighted gene co‐expression network analysis to explore some modules related to lymph node metastasis. Gene Ontology analysis and pathway enrichment analysis were conducted for functional annotation, and a protein‐protein interaction network was built. Samples from the International Cancer Genomics Consortium database were used as a validation set. The turquoise module showed the most relevance with lymph node metastasis. Functional annotation showed that biological processes and pathways were mainly related to activation of the processes of cell cycle and mitosis. Four hub genes were selected: CKAP2L, CDCA8, ERCC6L and ARPC1A. Further validation showed that the four hub genes well‐distinguished tumour and normal tissues, and they were good biomarkers for lymph node metastasis of PCa. In conclusion, the identified hub genes facilitate our knowledge of the underlying molecular mechanism for lymph node metastasis of PCa.

## 1 INTRODUCTION

Prostate cancer (PCa) is the fifth principal cause of death and also the second most frequent cancer in males all over the world.^1^ More than 15% of PCa patients harbour lymph node metastasis at radical prostatectomy.^2^ Lymph node metastasis is a complicated process in which cancer cells leave the primary tumour site through the lymphatic system and then establish a secondary tumour site in lymph nodes.^3^ These individuals have a higher risk of recurrence after primary treatment and usually suffer a poor prognosis.^4^ Therefore, predicting the occurrence of lymph node metastasis is of vital clinical significance. At the present day, several nomograms and indexes have been used to predict the occurrence of lymph node metastasis in PCa patients.^5^, ^6^ However, most of them are based on traditional biopsies or imaging‐based diagnoses and have limited accuracy and sensitivity in a way.^7^

In the past decade, an increasing number of microarray and next‐generation sequencing technologies have been used to explore novel therapeutic targets and prognostic biomarkers for various cancers, which also provide a good method to explore potential molecular biomarkers for lymph node metastasis of PCa.^8^ Weighted gene co‐expression network analysis (WGCNA) is an algorithm for weighted correlation network analysis, as well as a data exploratory tool or a gene screening method to explore clusters of highly correlated genes.^9^ It has been widely used to finding hub genes in many kinds of cancers. In this study, we used this algorithm to identify relevant modules and hub genes for lymph node metastasis of PCa, so that we can better understand the underlying molecular mechanism.

## 2 MATERIALS AND METHODS

### 2.1 Data collection and pre‐processing

**Figure 1:**
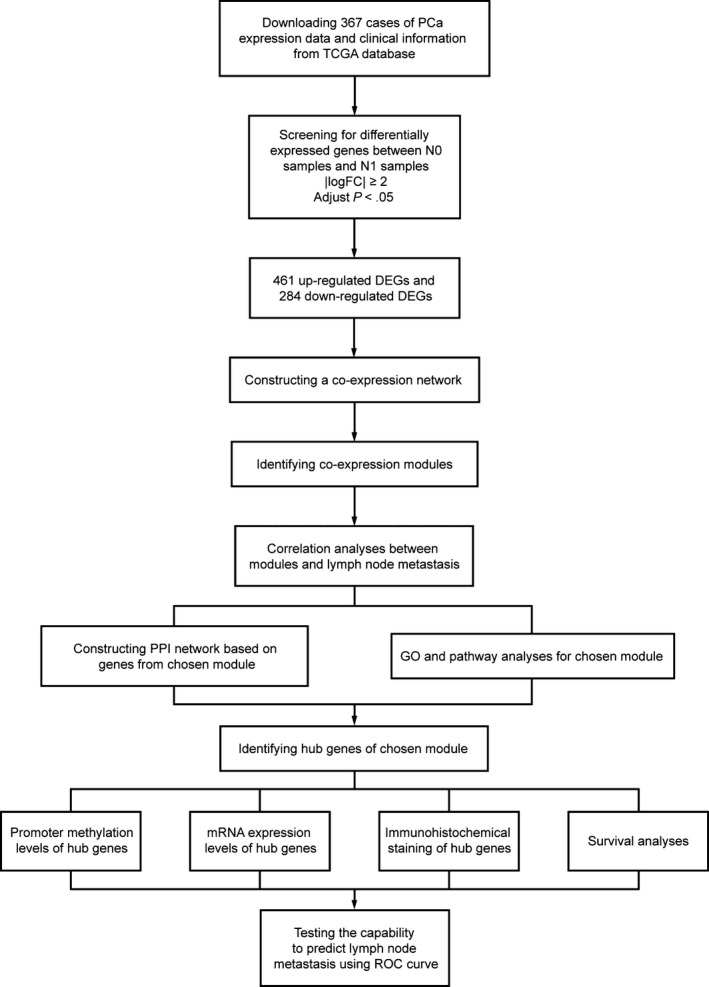
Flow chart detailing the study design and samples at each stage of the analysis

The workflow of the present study is shown in Figure 1. We downloaded expressing profiles of mRNA, including 367 PCa cases, from the cancer genome atlas (TCGA) database (https://portal.gdc.cancer.gov/). Clinicopathological characteristics of the 367 cases are summarized in Table 1. After screening the differentially expressed genes (DEGs) between PCa samples with and without lymph node metastasis, WGCNA was conducted to find the module relative to lymph node metastasis. Gene Ontology (GO) analysis and pathway enrichment analysis were conducted for functional annotation for selected module. We built protein‐protein interaction (PPI) network and selected hub genes according to the degree of connectivity. Then, online databases were used for further validation. Meanwhile, the additional independent data set including 279 cases of PCa samples from International Cancer Genomics Consortium (ICGC) database (https://dcc.icgc.org/) was used to perform survival analyses for the hub genes; receiver operating characteristic (ROC) curves of hub genes were plotted, and area under the curves (AUC) was calculated to evaluate their capability to distinguish a patient with lymph node metastasis or not. Clinicopathological characteristics of the 279 cases are summarized in Table 2.

**Table 1:** Clinicopathological characteristic of 367 patients with PCa from TCGA cohort

Clinicopathological characteristics	Value
Age, y	
Mean ± SD	61.37 ± 6.68
Range	41‐78
T stage, n(%)	
T2	117 (31.9)
T3	238 (64.8)
T4	8 (2.2)
Unknown	4 (1.1)
N stage, n(%)	
N0	291 (79.3)
N1	76 (20.7)
Survival, n(%)	
Yes	352 (95.9)
No	15 (4.1)

Abbreviations: PCa, prostate cancer; TCGA, the cancer genome atlas.

**Table 2:** Clinicopathological characteristic of 279 patients with PCa from ICGC cohort

Clinicopathological characteristics	Value
Age, y	
Mean ± SD	63.18 ± 7.05
Range	57‐82
T stage, n (%)	
T2	98 (35.1)
T3	168 (60.2)
T4	13 (4.7)
N stage, n (%)	
N0	199 (71.3)
N1	80 (28.7)
M stage, n (%)	
M0	258 (92.5)
M1	21 (7.5)
Survival, n (%)	
Yes	262 (93.9)
No	17 (6.1)

### 2.2 Differentially expressed genes screening

We screened the DEGs between PCa samples with and without lymph node metastasis in TCGA cohort by the ‘limma’ R package. Adjust *P*‐value < .05 and |logFC| ≥2 were set as the cut‐off criterion for a better accuracy and significance as described previously.^10^

### 2.3 Co‐expression network construction

Firstly, data pre‐processing and quality assessment were performed and WGCNA algorithm was used to construct a scale‐free co‐expression network for the DEGs. The Pearson's correlation matrices were calculated by average linkage method for all pairwise genes. After that, we constructed a weighted adjacency matrix by a power function $a_{\mathrm{mn}}={\left|c_{\mathrm{mn}}\right|}^{\beta }$ (*c_mn_* = Pearson's correlation between gene m and gene n; *a_mn_* = adjacency between gene *m* and gene *n*). *β* is a soft‐thresholding parameter that can emphasize strong correlations between genes and penalize weak correlations. We chose a proper power of *β* according to the mean connectivity. Then, the adjacency was transformed into a topological overlap matrix (TOM), and the corresponding dissimilarity (1‐TOM) was calculated.^11^ In order to classify genes with similar expression profiles into gene modules, average linkage hierarchical clustering was constructed according to the TOM‐based dissimilarity measure with a minimum size (gene group) of 30 for the genes dendrogram.

### 2.4 Identification of module associated with lymph node metastasis

We used two methods to identify modules relevant to clinical features of PCa. Gene significance (GS) was defined as the log10 transformation of the *P*‐value (GS = lg*P*) in the linear regression between gene expression and the clinical features. Module significance (MS) was defined as the average GS for all the genes in a module. The module with the absolute MS ranked first among all the selected modules was considered as the one related with the certain clinical feature. Module eigengene (ME) was considered as the major component in the principal component analysis for each gene module, and the expression patterns of all genes could be summarized into a single characteristic expression profile within a given module. We calculated the correlation between each ME and clinical features to identify the relevant module. The module with the maximal absolute MS among all the selected modules was usually considered as the one related with the certain clinical feature. Finally, the module highly correlated with the certain clinical feature was selected for further analysis.

### 2.5 Functional enrichment analysis

Webgestalt (http://www.webgestalt.org/) and Metascape (http://metascape.org/) are online databases providing a comprehensive set of functional annotation tools for researchers to better understand biological meaning behind large list of genes.^12^, ^13^ We uploaded genes in chosen module to perform GO analysis and pathway enrichment analysis. *P*‐value < .05 was considered statistically significant.

### 2.6 PPI network and hub genes selection

Search Tool for the Retrieval of Interacting Genes (STRING) is a biological database for constructing PPI networks, providing a system‐wide view of interactions between each member.^14^ Genes of selected module were mapped to STRING to explore their relationships with each other, and a combined score of >0.4 was set as the cut‐off criterion as described previously.^15^ Then, we established PPI network using Cytoscape software, which visually explores biomolecular interaction networks composed of proteins, genes and other molecules. The plug‐in Centiscape was used to search for the most important nodes in a network by calculating centrality parameters for each node.^15^ We selected hub genes based on the following criteria: high degree of connectivity; expression level was of statistically significant difference between samples with and without lymph node metastasis; overall survival was of statistically significant difference between samples with high and low gene expression level.

### 2.7 Validation of hub genes by online database

UALCAN (http://ualcan.path.uab.edu/) is a portal for facilitating tumour subgroup gene expression and survival analyses.^16^ Expression levels in mRNA and promoter methylation levels of hub genes were revealed using UALCAN. Additionally, the Human Protein Atlas (http://www. proteinatlas.org) was used for validation in immunohistochemistry aspect.

### 2.8 Survival analyses and ROC curve analyses of hub genes

Data set including 279 cases of PCa samples from ICGC database was used for validation. Patients from ICGC cohort were divided into two groups (high expression and low expression) according to a cut‐off value of mean expression of the hub genes. Then, survival analyses for hub genes were performed. The hazard ratio (HR) with 95% confidence intervals and log‐rank *P*‐value was calculated and displayed. Moreover, receiver operating characteristic (ROC) curves of hub genes were plotted and AUC was calculated with the ‘ROC’ R package to evaluate the capability of distinguishing a patient with lymph node metastasis or not.

### 2.9 Statistical analysis

Statistical analyses were conducted by SPSS version 22.0 (SPSS) and GraphPad Prism 5.0 (GraphPad Software). Survival curves were plotted by the Kaplan‐Meier method and compared by the log‐rank test.* P* < .05 was considered to be statistically significant.

## 3 RESULTS

### 3.1 DEGs screening

**Figure 2:**
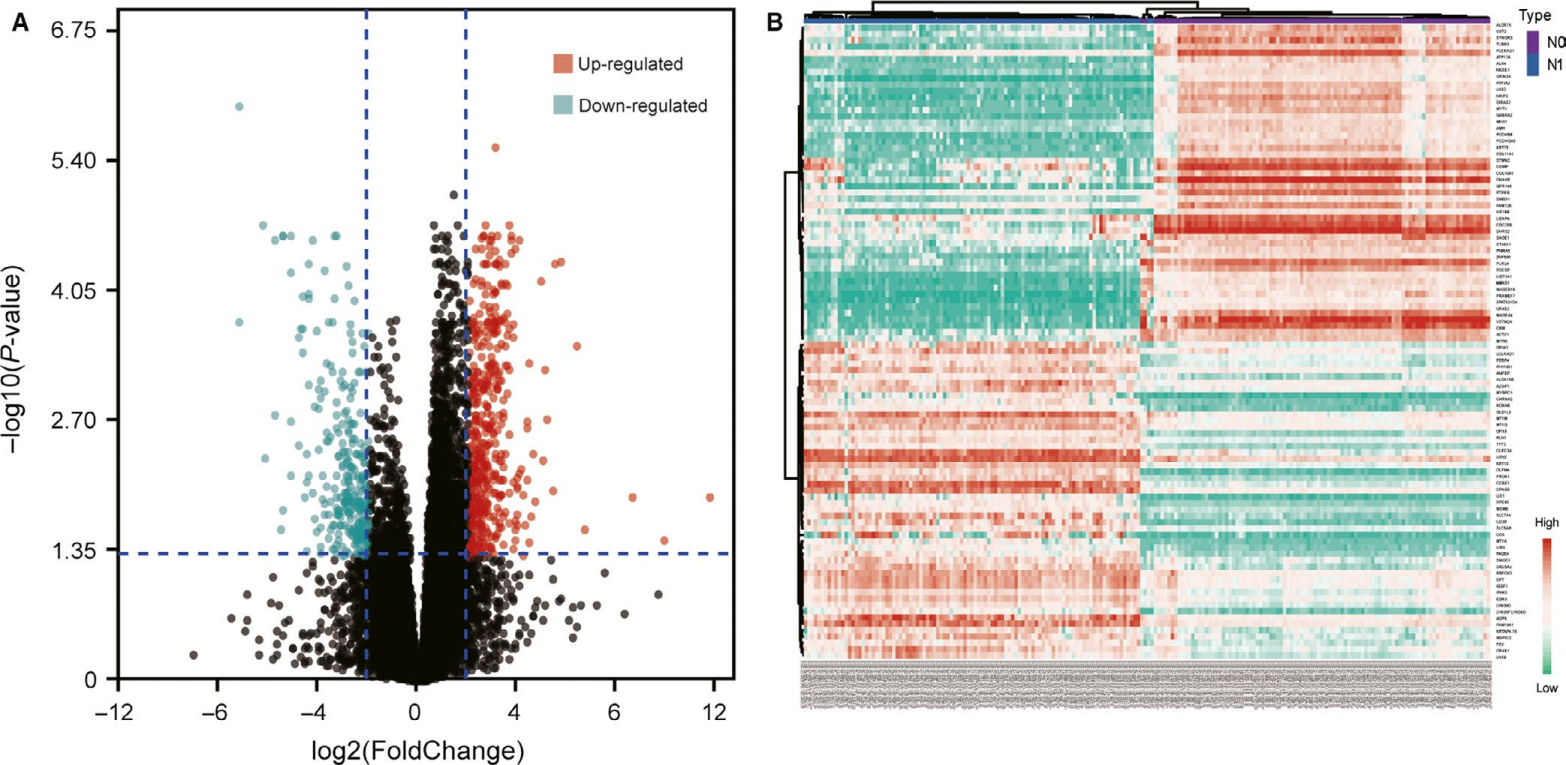
Selection of DEGs. A, Volcano plot of the DEGs (adjust *P*‐value < .05 and |logFC| ≥2 were set as the cut‐off criteria). B, Heat map of the top 100 DEGs (top 50 up‐regulated and down‐regulated genes). DEGs, differentially expressed genes

We obtained the expression data of 367 PCa samples after data pre‐processing and quality assessment. Under the threshold of adjust *P*‐value <.05 and |logFC| ≥2, a total of 745 DEGs (461 up‐regulated and 284 down‐regulated) were chosen for subsequent analysis. A volcano plot and heat map of DEGs are shown in Figure 2A,B, respectively.

### 3.2 Weighted co‐expression network construction and key modules identification

**Figure 3:**
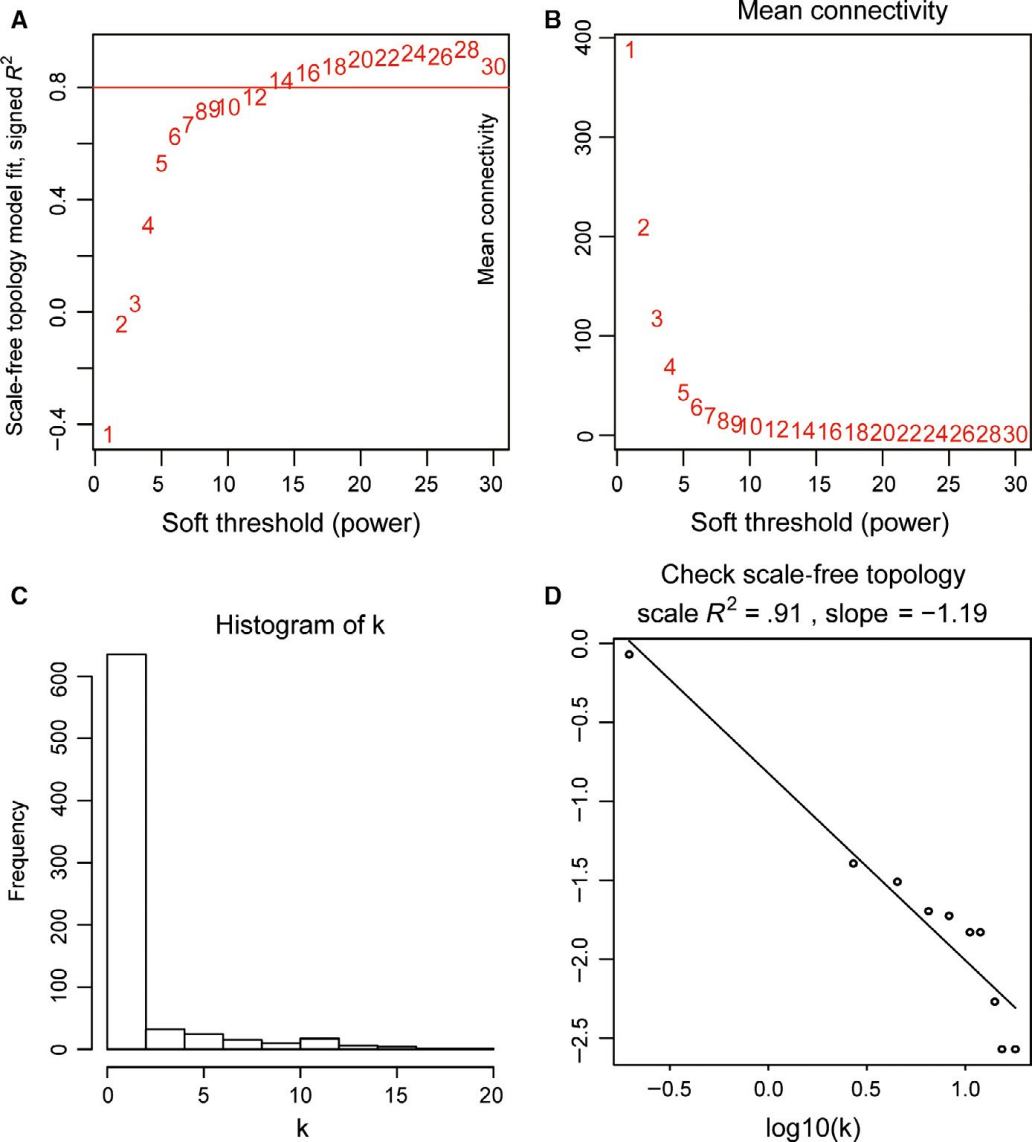
Determination of soft‐thresholding power in WGCNA. A, Analysis of the scale‐free fit index for various soft‐thresholding powers. B, Analysis of the mean connectivity for various soft‐thresholding powers. C, Histogram of connectivity distribution when *β* = 14. D, Check of scale‐free topology when *β* = 14. WGCNA, Weighted gene co‐expression network analysis

**Figure 4:**
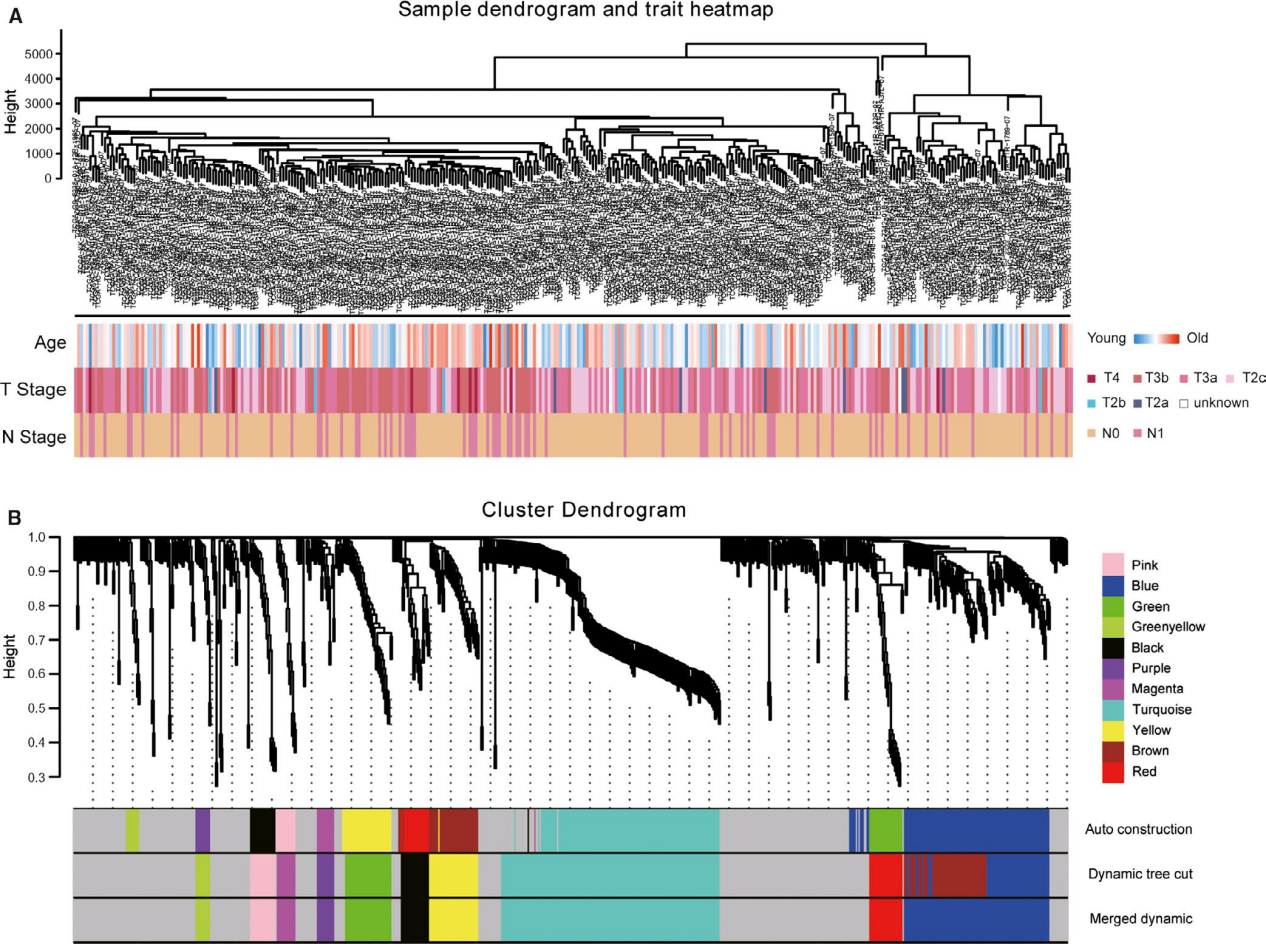
Sample dendrogram and clustering dendrogram of WGCNA. A, Sample dendrogram and corresponding clinical characteristics. B, Cluster dendrogram of 367 samples with eligible data. WGCNA, Weighted gene co‐expression network analysis

**Figure 5:**
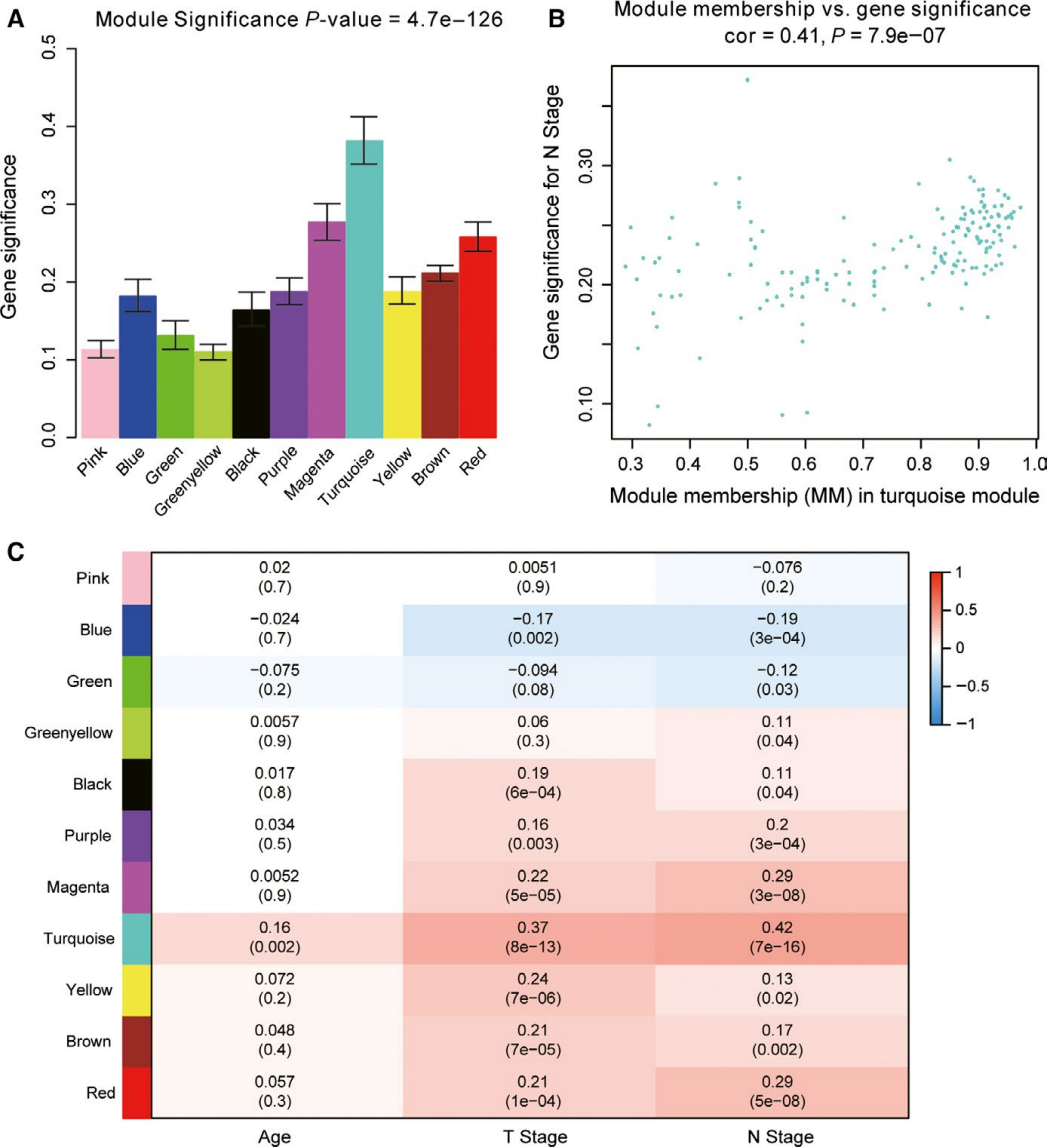
Identification of modules associated with clinical characteristics. A, Distribution of average gene significance and errors in the modules associated with lymph node metastasis in PCa. B, Scatter plot of module eigengenes in turquoise module. C, Heatmap of the correlation between module eigengenes and different clinical characteristics of PCa. PCa, prostate cancer

Firstly, we selected the power of *β* = 14 (scale free *R* ^2^ = .91) as the best soft‐thresholding parameter (Figure 3A,B); Figure 3C,D shows the positive result of the rationality test. After that, a sample dendrogram was constructed based on the similarity between the samples and the clinical characteristics of each sample are shown (Figure 4A). Finally, 11 modules were identified (Figure 4B). We used two methods to test the relevance between each module and lymph node metastasis. Modules with a higher MS value were considered to have more connection with the lymph node metastasis, and we found that the MS of the turquoise module was higher than those of any other modules (Figure 5A). Afterwards, the ME of the turquoise module showed a higher correlation with lymph node metastasis than other modules (Figure 5C). We finally identified the turquoise module, including 133 genes, was the module most relevant to lymph node metastasis in PCa (Figure 5B).

### 3.3 Functional enrichment analysis

**Figure 6:**
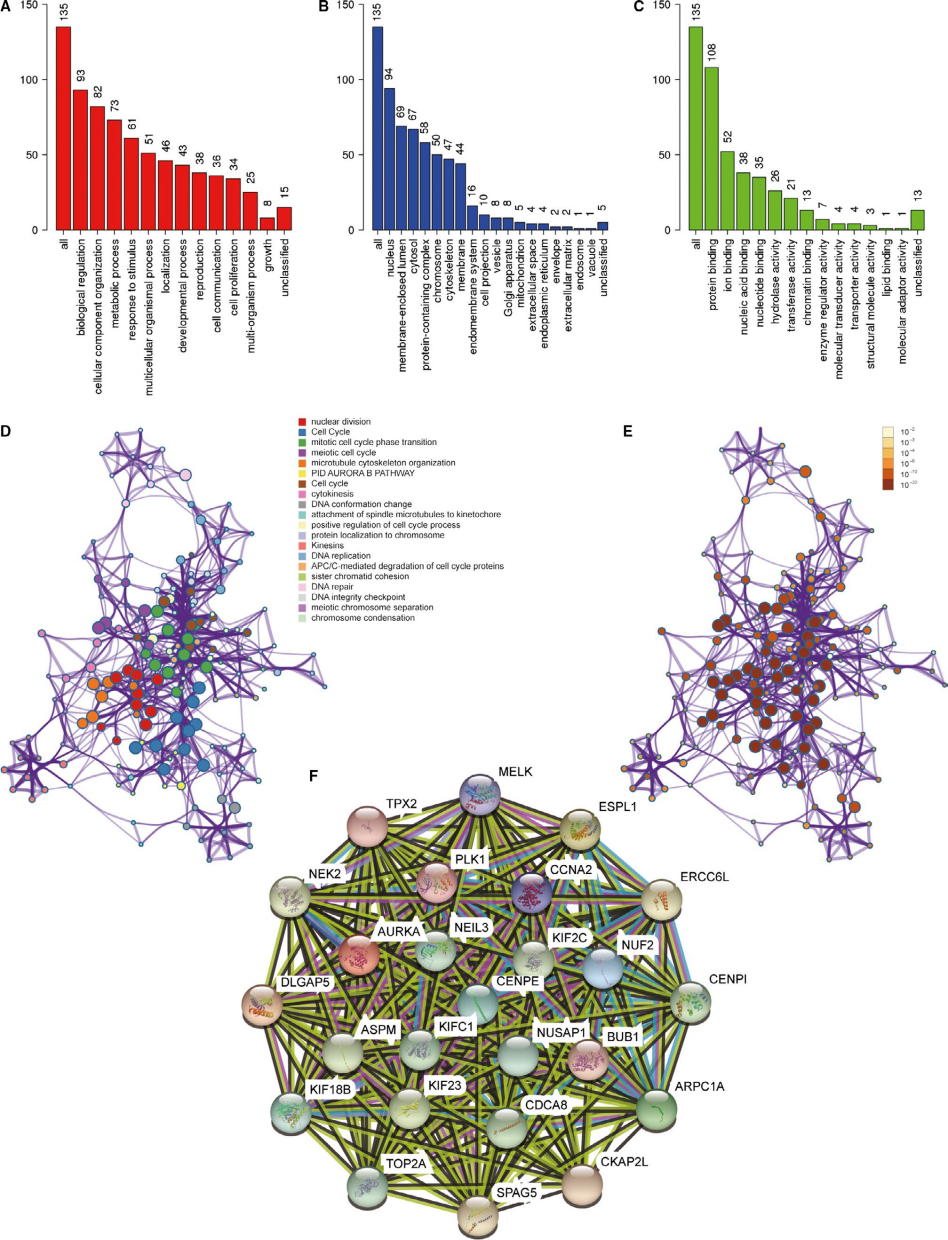
Functional enrichment analysis and construction of PPI network. A, GO analysis of biological process for genes in turquoise module. B, GO analysis of cellular component for genes in turquoise module. C, GO analysis of molecular function for genes in turquoise module. D, Functional enrichment analysis for genes in turquoise module. E, *P*‐value of each gene in the network. F, PPI network constructed using STRING. GO, gene ontology; PPI, protein‐protein interaction

We performed functional enrichment analysis to look for the biological processes and pathways relevant to turquoise module. GO analysis was conducted by the tool of Webgestalt. GO analysis of biological process revealed that genes in turquoise module were mainly involved in biological regulation, cellular component organization, metabolic process, response to stimulus and multicellular organismal process (Figure 6A). GO analysis of cellular component showed that these genes were mainly enriched in nucleus, membrane‐enclosed lumen, cytosol, protein‐containing complex and chromosome (Figure 6B). GO analysis of molecular function revealed that these genes were mainly involved in protein binding, ion binding, nucleic acid binding, nucleotide binding and hydrolase activity (Figure 6C). Metascape was used further investigate the relevant biological processes and pathways. The result showed that the biological processes and pathways were mainly related to the processes of cell cycle and mitosis (Figure 6D,E and Table 3).

**Table 3:** Functional enrichment analysis for genes in turquoise module

Category	Term	Count	%	*P*‐value	*q*‐value
GO biological processes	Nuclear division	51	38.35	1.47E‐55	3.06E‐51
Reactome gene sets	Cell cycle	54	40.60	1.04E‐49	5.38E‐46
GO biological processes	Mitotic cell cycle phase transition	37	27.82	8.01E‐29	1.39E‐25
GO biological processes	Meiotic cell cycle	27	20.30	2.47E‐27	3.66E‐24
GO biological processes	Microtubule cytoskeleton organization	34	25.56	7.84E‐26	9.04E‐23
Canonical pathways	PID AURORA B PATHWAY	13	9.77	1.69E‐20	9.22E‐18
KEGG pathway	Cell cycle	15	11.28	2.77E‐16	1.25E‐13
GO biological processes	Cytokinesis	16	12.03	1.71E‐15	6.57E‐13
GO biological processes	DNA conformation change	18	13.53	2.84E‐13	8.69E‐11
GO biological processes	Attachment of spindle microtubules to kinetochore	8	6.02	6.43E‐12	1.71E‐09
GO biological processes	Positive regulation of cell cycle process	16	12.03	9.44E‐12	2.45E‐09
GO biological processes	Protein localization to chromosome	10	7.52	2.78E‐11	6.95E‐09
Reactome gene sets	Kinesins	9	6.77	3.52E‐11	8.71E‐09
GO biological processes	DNA replication	15	11.28	3.68E‐11	8.88E‐09
Reactome gene sets	APC/C‐mediated degradation of cell cycle proteins	10	7.52	4.51E‐11	1.04E‐08
GO biological processes	sister chromatid cohesion	9	6.77	6.51E‐11	1.44E‐08
GO biological processes	dna repair	19	14.29	2.15E‐10	4.43E‐08
GO biological processes	DNA integrity checkpoint	11	8.27	1.17E‐09	2.12E‐07
GO biological processes	Meiotic chromosome separation	6	4.51	4.04E‐09	7.06E‐07
GO biological processes	Chromosome condensation	7	5.26	6.85E‐09	1.18E‐06

Abbreviation: GO, gene ontology; KEGG, Kyoto Encyclopedia of Genes and Genomes.

### 3.4 Identification of hub genes and analysis of modules from PPI networks

A PPI network was constructed by STRING (Figure 6F). Finally, we selected four hub genes for further discussion. They were CKAP2L (Cytoskeleton Associated Protein 2 Like), CDCA8 (Cell Division Cycle Associated 8), ERCC6L (ERCC Excision Repair 6 Like, Spindle Assembly Checkpoint Helicase) and ARPC1A (Actin Related Protein 2/3 Complex Subunit 1A). These four genes were all of significantly clinical significance. The module membership values of these four hub genes were 0.92, 8 0.74, 0.80 and 0.93, respectively, which were of statistical significance. The scatter plot of module eigengenes in turquoise module was showed in Figure 5B. This result was basically consistent with PPI network.

### 3.5 Validation and efficacy evaluation of hub genes

**Figure 7:**
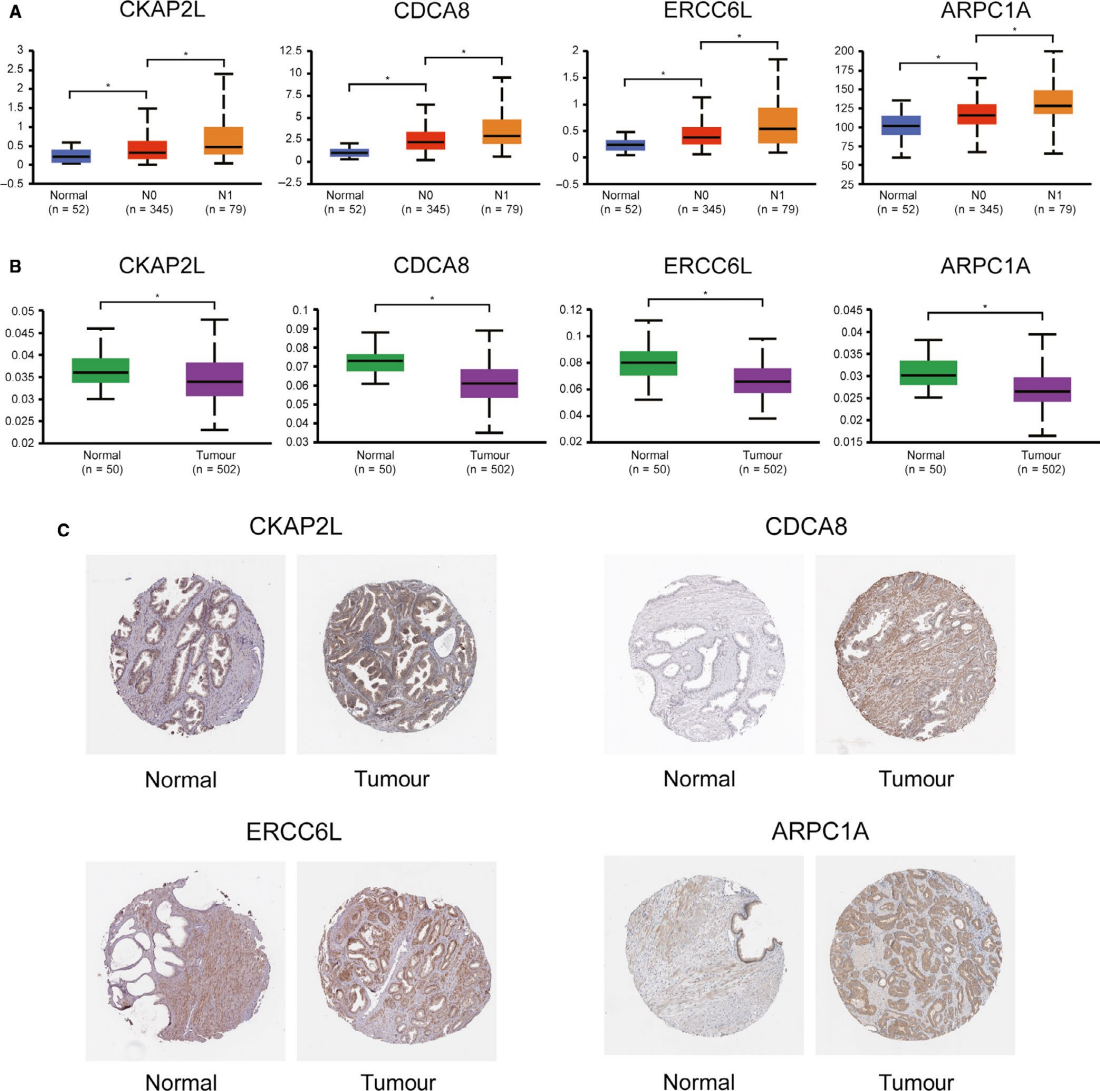
Validation of the hub genes using online databases. A, Expression levels of the hub genes in PCa and normal prostate tissues. B, Promoter methylation levels of the hub genes in PCa and normal prostate tissues. C, Immunohistochemical staining of the hub genes in PCa and normal prostate tissues

We used data set from the online database UALCAN for validation. These hub genes revealed higher expression levels in samples with lymph node metastasis than those without lymph node metastasis, let alone those in normal tissues (Figure 7A). Besides, compared with the normal tissues, these hub genes revealed lower levels of promoter methylation in PCa tissues (Figure 7B). Additionally, immunohistochemistry staining from the Human Protein Atlas database revealed that protein levels of the hub genes were significantly higher in PCa tissues compared with normal tissues (Figure 7C).

**Figure 8:**
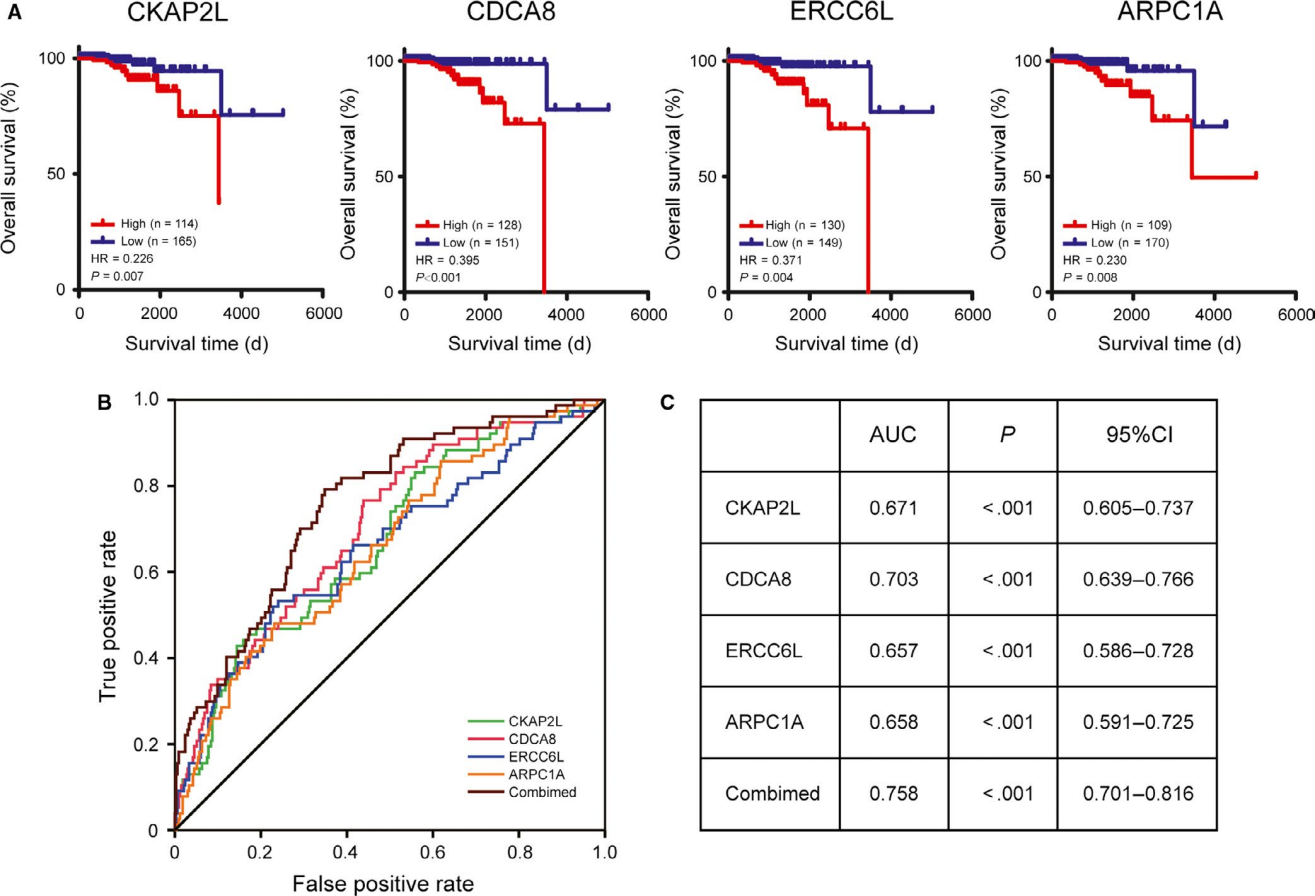
Validation of the hub genes based on the ICGC cohort. A, Overall survival between patients with high and low expression of the four hub genes. B, ROC curves of the four hub genes to evaluate their capability in predicting lymph node metastasis of PCa. C, AUC and 95% confidence interval of the four hub genes

A data set including 279 cases of PCa samples from the ICGC database was used for validation. It was found that increased expression levels of CKAP2L (HR 0.226 [0.082‐0.465], *P* = .007) were associated with poor overall survival in PCa patients, as well as CDCA8 (HR 0.395 [0.125‐0.626], *P* < .001), ERCC6L (HR 0.371 [0.108‐0.593], *P* = .004), and ARPC1A (HR 0.230 [0.090‐0.477], *P* < .001) (Figure 8A). In addition, ROC curve analyses were performed to evaluate the capability of the hub genes to distinguish a patient with lymph node metastasis or not. AUC values for these hub genes were greater than 0.5, and the AUC value combined all the hub genes revealed the best value (0.758 [0.701‐0.816], *P* < .001; Figure 7B,C).

## 4 DISCUSSION

Lymph node metastasis indicates poor prognosis for PCa patients.^3^, ^4^ Therefore, elucidation of the molecular mechanism of lymph node metastasis is crucial to understand the disease progression and also to develop novel therapeutic targets. Cytokines secreted by tumour cells will promote the genesis of tumour‐associated lymphatic vessels, which form an easy passage for cancer cells to lymph nodes and distant organs. In addition, tumour cells can be stimulated by cytokines produced by the lymphatic vessels, promoting chemotactic diffusion of tumour cells into the lymphatics.^17^, ^18^, ^19^ This is the basic process of lymph node metastasis; however, the exact mechanisms have not been well studied.^17^ In this study, we applied WGCNA to identify the key modules and hub genes in lymph node metastasis of PCa. Functional enrichment analysis was performed, and PPI network was built to further explore the biological significance. The additional independent data set was used to confirm the reliability of the results. This study provides novel insights that will help to explain the mechanism of lymph node metastasis in PCa at the molecular level, and the hub genes identified might act as potential biomarkers as well as therapeutic targets for precise diagnosis and treatment in the future.

In the present study, the turquoise module identified by WGCNA was the most significantly related module to lymph node metastasis of PCa. In functional enrichment analysis conducted based on genes in turquoise module, we found that these genes were mainly related to the process of cell cycle and proliferation. Specifically, pathway analysis indicated that 51 up‐regulated genes were significantly enriched in nuclear division and 54 up‐regulated genes were enriched in cell cycle. The results were in accordance with previous reports that dysregulation of the cell cycle progression and uncontrolled cell proliferation were the hallmarks of cancer.^20^ The result of functional enrichment analysis revealed that dysregulation of the key genes in the processes of cell cycle and proliferation would promote lymph node metastasis of PCa.

Owing to the importance of turquoise module in lymph node metastasis of PCa, we screened the hub genes of this module. Four hub genes were selected according to the degree of connectivity in PPI network. CKAP2L, also known as Radmis, is a microtubule‐associated protein that appears at the mitotic phase and participates in the cell division of neural progenitor cell.^21^ Besides, CKAP2L has been proved to be a vital component of centrosome and is situated in the spindle, the midbody and the spindle pole.^22^ A recent study revealed that high expression of CKAP2L promoted the invasion of lung cancer through MAPK signalling pathway and was associated with poor prognosis.^23^ In addition, the oncogenic nature of CKAP2, which is an important paralog of CKAP2L, has also been illustrated in ovarian cancer, glioma and breast cancer.^24^, ^25^, ^26^ CKAP2L is a promising candidate gene that may affect the tumorigenesis and lymph node metastasis of PCa; however, more studies are required to confirm it. CDCA8, also called Borealin/Dasra B, is a member of the chromosomal passenger complex necessary for transmission of the genome during cell division.^27^ It plays a vital role in mitosis, intersecting chromosome segregation and cell division with cancer.^28^ Previous study revealed that CDCA8 was overexpressed in colorectal cancers and that loss of CDCA8 suppressed the growth of cancer cells and induced apoptosis.^28^ Furthermore, it was reported that high expression of CDCA8 was significantly associated with lymph node metastasis in melanoma.^29^ CDCA8, therefore, may work as a promoter in lymph node metastasis of PCa and have the potential to become a novel diagnostic and therapeutic factor for PCa. ERCC6L, also known as polo‐like kinase 1 (PLK1)‐interacting checkpoint helicase, has been demonstrated to be a development‐associated member of the SNF2 family.^30^, ^31^ It plays wide roles in regulating cell division, cell proliferation and other biological processes and is considered as a genetic marker in the development of tumours.^32^ It was reported that ERCC6L was relevant to disease progression and poor prognosis in many types of cancer, such as breast, renal and colorectal cancer.^33^, ^34^ High expression of ERCC6L plus its role in embryonic development and the involvement of remodelling centromeric chromatin remind us to hypothesize that it may play a key role in tumorigenesis. However, further studies are required to clarify the role of ERCC6L in lymph node metastasis of PCa. ARPC1A is a member of the actin‐related protein 2/3 (ARP2/3) complex family.^35^ The ARP2/3 complex takes part in the process of actin filament nucleation and depolymerization which are necessary for the formation of invasive pseudopodia in cancer cells.^36^ Previous studies have reported that components of the ARP2/3 complex were highly expressed in various tumours, including bladder, breast, gastric and lung cancers.^36^, ^37^, ^38^, ^39^ Consequently, ARPC1A is a promising candidate biomarker or predictor for lymph node metastasis of PCa when more studies confirm its value.

Analyses by UALCAN and the Human Protein Atlas online databases confirmed the function and the clinical significance of these hub genes. Besides, ROC curve analyses conducted based on the additional independent data set from ICGC database revealed that these hub genes did well in predicting lymph node metastasis of PCa, especially when combining all of them. Therefore, these hub genes may become potential biomarkers for predicting lymph node metastasis of PCa.

In conclusion, we used a series of bioinformatics analysis methods to identify the key genes involved in lymph node metastasis of PCa. Our results provide a more detailed molecular mechanism for lymph node metastasis of PCa, shedding light on the potential biomarkers and therapeutic targets. However, the interacting mechanism and function of genes need to be confirmed in further experiments.

## CONFLICT OF INTEREST

The authors confirm that there are no conflicts of interest.

## AUTHORS’ CONTRIBUTIONS

Qing‐Shui Zheng and Ye‐Hui Chen designed the work; Zhi‐Bin Ke, Ru‐Nan Dong, Peng Huang and Xiao‐Dong Li carried out the experiments and analysed the data with the guidance of Qing‐Shui Zheng; Ning Xu, Shao‐Hao Chen, Ting‐Ting Lin and Hai Cai prepared the manuscript. All authors drafted or critically revised the manuscript for important intellectual content and approved the final version of the manuscript.

## Data Availability

The data sets used and/or analysed in this study are available from the corresponding author on reasonable request.
